# Supplementary material for: Genome-Wide Identification and Functions against Tomato Spotted Wilt Tospovirus of PR-10 in Solanum lycopersicum
Source: Int J Mol Sci. 2022 Jan 28;23(3):1502. doi: 10.3390/ijms23031502 (PMC8835967; doi:10.3390/ijms23031502)
Supplement: Supplementary file 1 [file ijms-23-01502-s001.zip › ijms-1562724-supplementary.pdf]

International Journal of *Molecular Sciences*

MDPI

Table S1. Primers for PR-10, TSWV-cp protein, *Sw-5b*, *actin* gene.

PR-10 -F	GGTTCTTGATTTTGATAGCC
PR-10 -R	GATTCCAATTTGTCACCAAG
TSWV-cp-F	TCTGTGAGGCTTGCCATAAT
TSWV-cp-R	AGCATACTCTTTCCCTTTCT
SW-5b -F	TTGTCGTCGGAACCTGTA
SW-5b -R	TGATGAGGAGGTAGTGGGTT
SlActin-F	AGGCACACAGGTGTTATGGT
SlActin -R	AGCAACTCGAAGCTCATTGT
